# Seroprevalence of anti-diphtheria toxoid antibody and implications for vaccination policy in Vietnam’s South-central coast: a cross-sectional study

**DOI:** 10.1186/s12879-024-09688-0

**Published:** 2024-08-12

**Authors:** Hoang Thieu Le, Thai Hung Do, The Anh Dao, Tien Thanh Hoang, Bao Trieu Nguyen, Thuy Lien Le, Dinh Luong Nguyen, Lay-Myint Yoshida, Xuan Huy Le, Hong Quan Le, That Toan Ton, Min Jin Ha

**Affiliations:** 1 Pasteur Institute in Nha Trang, Nha Trang, Khanh Hoa Vietnam; 2 Department of Pediatric Infectious Diseases Institute of Tropical Medicine, Nagasaki University, Nagasaki, Japan; 3 Khanh Hoa Province Health Department, Nha Trang, Khanh Hoa Vietnam; 4 Nha Trang Center for Disease Control, Nha Trang, Khanh Hoa Vietnam; 5 Department of Health Informatics and Biostatistic, Graduate School of Public Health, Yonsei University, Seoul, Republic of Korea

**Keywords:** Seroprevalence, Anti-diptheria toxoid antibody, Vietnam

## Abstract

**Background:**

Diphtheria is a re-emerging infectious disease and public health concern worldwide and in Vietnam with increasing cases in recent years. This study aimed to assess the anti-diphtheria toxoid antibodies status in Khanh Hoa Province and identify factors contributing to the vaccination policy in the south-central coast of Vietnam.

**Methods:**

This was a cross-sectional study to evaluate the seroprevalence of anti-diphtheria toxoid antibodies among 1,195 participants, aged 5 – 40 years in Khanh Hoa Province, Vietnam. Immunoglobulin G antibody levels against diphtheria were detected using a commercial anti-diphtheria toxoid enzyme-linked immunosorbent assay (SERION ELISA classic Diphtheria Immunoglobulin G) and were categorized following the World Health Organization guidelines.

**Results:**

The mean anti-diphtheria toxoid antibody levels were 0.07 IU/ml (95% Confidence Interval: 0.07–0.08). Anti-diphtheria toxoid antibody levels were found to be associated with age and history of diphtheria vaccination. The 5–15 years age group had the highest levels (0.09 IU/ml), while the older age group had the lowest antibody level (*p* < 0.001). Individuals who received three doses (adjusted Odds ratio: 2.34, 95%CI: 1.35 – 4.07) or 4^+^ doses (adjusted Odds ratio: 2.45, 95%CI: 1.29 – 4.64) had a higher antibody level compared to those who received only one dose regardless of age.

**Conclusion:**

It is crucial to promote routine vaccination coverage to over 95% for children under one year of age with three primary doses of the diphtheria-containing vaccine, including additional doses at 18 months and 7 years of age. Booster doses should be promoted and administered to adolescents and adults every 10 years.

**Supplementary Information:**

The online version contains supplementary material available at 10.1186/s12879-024-09688-0.

## Background

Diphtheria is a bacterial disease caused by the* Corynebacterium *species* C. diphtheriae, C. ulcerans, *and* C. pseudotuberculosis *form the* C. diphtheriae* group with a fatality rate ranging from 5 to 10% [1]. According to the World Health Organization (WHO), between 2011 and 2021, there were 101,699 diphtheria cases, of which over 60,000 were reported in Southeast Asia, followed by Africa, the Eastern Mediterranean, the Americas, the Western Pacific, and Europe [2]. Therefore, diphtheria is an important re-emerging infectious disease and public health concern worldwide. The WHO released recommendations for diphtheria vaccination in August 2017, including three doses of primary series for children under one year of age and other booster doses to provide immunity for all age groups [3].

In Vietnam, from 1974 to 2011, a total of 34,147 diphtheria cases were recorded [2]. There have been signs of a resurgence in the number of diphtheria cases in recent years, the number of cases has increased from 13 cases in 2018 to 237 cases in 2020 [2]. In 2020, in the South-central coast region of Vietnam, there were 28 confirmed cases of diphtheria with the majority reported in children aged five years and older. Among these cases, up to 68% (19/28) had previously received 3–4 doses of vaccines that contained diphtheria toxoid vaccine [4]. Since 1984, Vietnam's Expanded Program for Immunization (EPI) has given three doses of a diphtheria vaccine to children aged 2–4 months. Additional doses are given to 18-month-olds in all provinces, including Khanh Hoa, and to 7-year-olds in some risk areas through school and community initiatives. Within the EPI in Vietnam, the Diphtheria, Pertussis, Tetanus (DPT) vaccine in Vietnam combines diphtheria toxoid, tetanus toxoid, and inactivated pertussis bacteria, all adsorbed on an aluminum phosphate adjuvant. This vaccine was administered as a basic series dose for children under one year of age from 1984 to 2009 and for children at 18 months as an additional dose starting from 1984. The 5-in-1 vaccine, which protects against diphtheria, whooping cough, tetanus, hepatitis B, and Haemophilus influenzae type b (DPT-VGB-Hib), has been administered to children under one year of age since 2010. On average, the coverage of the diphtheria vaccine for children under one year of age was 88% from 1984 to 2021. In Khanh Hoa province, this rate is approximately 94% [4]. It raises the question of whether the level of the anti-diphtheria toxoid antibody in the community is enough to protect the population from diphtheria, especially in the South-central coastal province of Vietnam.

Our study aimed to provide evidence of the anti-diphtheria toxoid antibody status in the general population in the South-central coast of Vietnam and identify factors contributing to the vaccination policy for diphtheria prevention.

## Materials and methods

### Study population and area

Khanh Hoa is a province on the South-central coast with ~ 1.2 million people, of which 42.4% are urban and 57.6% are rural residents [5]. This province shares a border with the Central Highlands and is located in the middle of the South-central coast area. All eight districts/cities within the province were included in this study.

### Study design and sampling

This cross-sectional study assessed the seroprevalence of anti-diphtheria toxoid antibodies and the associated factors. This study employed probability proportional to size (PPS) sampling to select 30 communes/wards out of 136 communes/wards across eight districts/cities. This selection was accomplished by initially listing all 136 communes/wards, each accompanied by its cumulative population, and subsequently dividing the cumulative population by the number of clusters to be sampled (i.e., 30 communes/wards). This calculation yielded the sampling interval (k), and the first cluster was selected by choosing a random number between one and k. Subsequent clusters were chosen by adding multiples of k (2 k, 3 k, etc.) until a total of 30 clusters corresponding to 30 communes/wards were identified and included in the study.

Following the selection of these clusters, one subgroup (village) was randomly selected from each of the 30 clusters using a simple random sampling approach. Subsequently, within each subgroup, one residential area was randomly chosen. Households were systematically selected through door-to-door visits. Within each household, two participants were chosen randomly from a list of household members in the age group 5- 40 years.

The sample size was *n* = 1.200 blood samples/participants, calculated by using the single-proportion sample size formula [6] with *p* = 0.5 to estimate for the proportion of people have anti-diphtheria antibodies in the community, maximize the sample size due to no previous referenced study in Khanh Hoa province. The design effect (DE) is 3 to adjust the required sample size for cluster sampling.

### Serological assay and measurement

Levels of IgG antibody against diphtheria were determined using a commercial anti-diphtheria toxoid enzyme-linked immunosorbent assay (SERION ELISA classic Diphtheria IgG). The collected sera were stored at -80 °C in the Pasteur Institute in Nha Trang until testing. Applying the WHO guidelines for categorizing antibody levels [1], anti-diphtheria toxoid antibody levels < 0.01 IU/ml were considered as “No protection”, levels of 0.01—< 0.1 IU/ml were considered as “Partial protection” and levels of ≥ 0.1 IU/ml were considered as “Full protection”.

### Ethical approval

The procedures, participation agreement, participant selection documents, and related materials have been reviewed and approved by the Institutional Review Board on Biomedical Research of the Pasteur Institute in Nha Trang and the Institutional Review Board on Biomedical Research of the Khanh Hoa Provincial Department of Health.

### Statistical analysis

The categorical variables were described as (relative) frequency and continuous variables as mean and standard deviation (SD). Geometric mean concentrations (GMC) of antibody levels and 95% confidence intervals (CI) were calculated. The GMC of diphtheria toxoid antibodies between the factor groups were compared using a one-way ANOVA. Multiple logistic regression was used to analyze the factors associated with the dichotomized antibody levels of no/partial protection and full protection. All statistical analyses were performed using R version 4.2.2, with a significance level of 0.05, for all testing procedures.

## Results

**Fig. 1:**
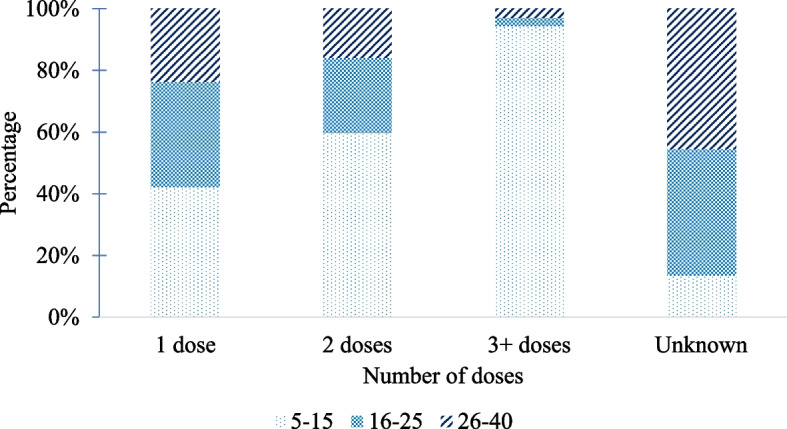
Distribution of age group by number of doses of diphtheria component vaccine (DPT,Td, pentavalent vaccine, hexavalent vaccine)

**Fig. 2:**
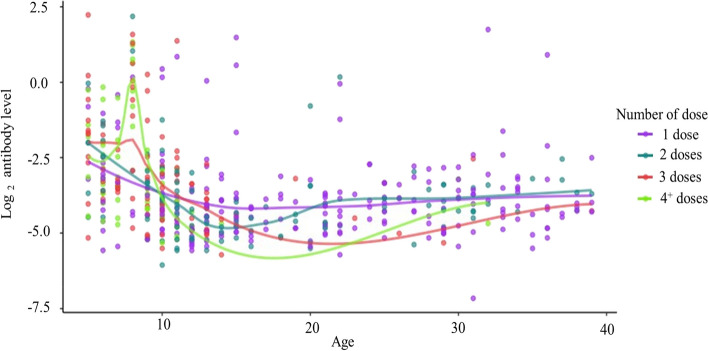
Distribution of antibody levels over age by diffenrent number of doses of vaccine

Out of 1,200 participants required, a total of 1,195 participants in the age group of 5 -40 years in eight districts/cities in Khanh Hoa Province, Vietnam, were recruited. The mean age was 22.3 ± 10.3; the majority of the participants were female (63.4%), and over 93.0% of the participants belonged to the Kinh ethnicity (Table 1). Of the participants, 40.2% had vaccine records and were administered at least one dose of the vaccine, including the DPT; Tetanus, diphtheria (Td); pentavalent, or hexavalent vaccine (Table 1). The majority of the participants who were administered at least three doses, were mostly in the age group 5 -15 years, whereas the majority of unknown status belonged to the older age groups (16 – 25 years and 26 – 40 years) (Fig. 1); this is mostly due to the absence of a vaccine record. The GMC of antibody levels of the participants was 0.07 IU/ml (95%CI: 0.07–0.08). The participants were categorized as “No protection” (0.1%), “Partial protection” (73.2%), and "Fully protection” (26.7%). The high level of antibody (> 0.1 IU/ml) was distributed in the age group 5–15 years, a high proportion of whom were administered 3 or 4^+^ doses of the vaccine (Fig. 2). The Fig. 2 illustrates the relationship between age and antibody levels on a log2 scale. Using a log2 scale, the y-axis spreads out more from negative to positive values, which helps to better distinguish differences in trends across various dose levels. The lines in the figure represent the different dose levels received by study participants, with each line fitted using LOWESS (Locally Weighted Scatterplot Smoothing).

**Table 1:** The anti-diphtheria toxoid antibody level

Variables	n	%	Anti-diphtheria toxoid antibody level GMC (IU/ml)		
			**mean**	**95%CI**	***p*-value¶**
**Age**					
(Mean ± SD: 22.3 ± 10.3)					** < 0.001**
5-15	394	33	0.09	0.08 – 0.1	
16–25	293	24.5	0.06	0.06 – 0.07	
26–40	508	42.5	0.07	0.07 – 0.08	
**Gender**					0.969
Male	437	36.6	0.07	0.07 – 0.08	
Female	758	63.4	0.07	0.07 – 0.08	
**Race**					0.672
Kinh (majority)	1,117	93.5	0.07	0.07 – 0.08	
Ethnic minority	78	6.5	0.07	0.06 – 0.09	
**Occupation**					**0.011**
Farmer/Fisher	39	3.3	0.06	0.05 – 0.07	
Engineer/Trader/Freelancer/Driver	339	28.4	0.07	0.07 – 0.08	
Officer	138	11.5	0.07	0.06 – 0.07	
Student	502	42	0.08	0.07 – 0.09	
Unemployment	118	9.9	0.07	0.06 – 0.08	
Others^a^	59	4.9	0.1	0.07 – 0.12	
**Participant’s education**					** < 0.001**
Illiteracy	37	3.1	0.14	0.09 – 0.2	
No school	133	11.1	0.13	0.11 – 0.17	
Primary school	289	24.2	0.08	0.07 – 0.09	
Secondary school	273	22.8	0.06	0.06 – 0.07	
High school	207	17.3	0.06	0.06 – 0.07	
Intermediate/College or higher	256	21.4	0.07	0.06 – 0.07	
**Number of children < 15 living in one house**					**0.034**
≤ 2 children	1,057	88.5	0.07	0.07 – 0.08	
˃ 2 children	138	11.5	0.09	0.07 – 0.11	
**Distance from home to nearest health centers**					0.243
< 1 km	265	22.2	0.07	0.06 – 0.08	
1- < 5 km	895	74.9	0.08	0.07 – 0.08	
≥ 5 km	35	2.9	0.09	0.06 – 0.12	
Residence					0.39
Rural area	199	16.7	0.08	0.07 – 0.09	
Urban area	996	83.3	0.07	0.07 – 0.08	
**History of diphtheria vaccination**^**b**^					** < 0.001**
1 dose	224	18.7	0.07	0.06 – 0.08	
2 doses	109	9.1	0.07	0.06 – 0.08	
3 doses	90	7.5	0.12	0.09 – 0.15	
4^+^ doses	58	4.9	0.13	0.1 – 0.18	
Unknown	714	59.7	0.07	0.07 – 0.07	
**A history of travel to and residence in a diphtheria epidemic area**					**0.024**
Yes	173	14.5	0.07	0.06 – 0.07	
No	1,022	85.5	0.08	0.07 – 0.08	

^¶^One-way Anova test

^a^Others (Carpenter, hairdresser, receptionist, teacher), *SD* Standard deviation

^b^Including *DPT, Td, pentavalent vaccine, hexavalent vaccine*

There was a significant difference (*p* < 0.001) in antibody levels in the age group with high GMC (0.09 IU/ml) in the group from 5–15 years of age compared to group from 16–25 years (0.06 IU/ml) and group from 26–40 years (0.07 IU/ml). The history of vaccination has a significant difference in antibody levels with high GMC in those who were administered 4^+^ doses or 3 doses compared to 2 doses or 1 dose with GMC at 0.13 IU/ml, 0.12 IU/ml, 0.07 IU/ml, and 0.07 IU/ml respectively. Significant differences were also found in antibody levels according to the participants’ education (*p* < 0.001), number of children aged < 15 years living in the house (*p* = 0.034), and history of traveling to epidemic areas (*p* = 0.024) (Table 1).

The multiple logistic regression model showed that there was a significant difference in antibody levels in the age group of 5–15 years compared with the age group of 16–25 years and the age group 26 – 40 years, respectively (*p* < 0.001). In addition, there was a significant difference in antibody levels with the history of vaccination: the participants who were administered three doses (adjusted OR: 2.34, 95%CI: 1.35 – 4.07, *p* = 0.002) or 4^+^ doses of vaccine (adjusted OR: 2.45, 95%CI: 1.29 – 4.64, *p* = 0.006) had antibody levels more than twice compared to those who received one dose (Table 2). Moreover, in the subgroup analyses by age, there is also a significant difference in antibody levels with a number of vaccine doses in the age group of 5 – 15 years with those who got three doses (adjusted OR: 2.33, 95%CI: 1.21 – 4.49, *p* = 0.011) or 4^+^ doses (adjusted OR: 2.37, 95%CI: 1.14 – 4.92, *p* = 0.021) having antibodies levels more than twice compare to those who got one dose (Table 3).

**Table 2:** Multiple logistic regression on factors associated with anti-diptheria toxoid antibody category

Variables	No protection or Partial protection n(%)	Full protection n(%)	aOR (95%CI)	*p-*value
**Age**				
5–15	253 (64.2)	141 (35.8)	Ref	
16–25	249 (85.0)	44 (15.0)	0.45 (0.29–0.71)	** < .001**
26–40	374 (73.6)	134 (26.4)	0.88 (0.59–1.31)	0.536
**Gender**				
Male	320 (73.2)	117 (26.8)	Ref	
Female	556 (73.4)	202 (26.7)	1.02 (0.76–1.37)	0.9
**Race**				
Kinh (majority)	824 (73.8)	293 (26.2)	1.4 (0.62–3.17)	0.414
Others (ethnic minority)	52 (66.7)	26 (33.3)	Ref	
**Residence**				
Urban area	740 (74.3)	256 (25.7)	Ref	
Rural area	136 (68.3)	63 (31.7)	1.06 (0.68–1.63)	0.807
**Income**				
Low income	84 (68.9)	38 (31.2)	1.09 (0.67–1.78)	0.723
High/middle income	792 (73.8)	281 (26.2)	Ref	
**Number of people living in one house**				
≤ 4 people	504 (74.3)	174 (25.7)	Ref	
˃ 4 people	372 (72.0)	145 (28.0)	1.02 (0.76–1.37)	0.885
**Number of children < 15 living in one house**				
≤ 2 children	790 (74.7)	267 (25.3)	Ref	
˃ 2 children	86 (62.3)	52 (37.7)	1.38 (0.89–2.14)	0.151
**Distance from home to the nearest healthcare center**				
< 1 km	206 (77.7)	59 (22.3)	Ref	
1- < 5 km	639 (73.2)	234 (26.8)	1.35 (0.96–1.89)	0.083
≥ 5 km	31 (54.4)	26 (45.6)	3.51 (1.61–7.64)	**0.002**
**Heard about diphtheria**				
Yes	257 (78.1)	72 (21.9)	Ref	
No	619 (71.5)	247 (28.5)	1.13 (0.81–1.57)	0.468
**History of diphtheria vaccination**				
1 dose	172 (76.8)	52 (23.2)	Ref	
2 doses	82 (75.2)	27 (24.8)	1.06 (0.61–1.85)	0.837
3 doses	49 (54.4)	41 (45.6)	2.34 (1.35–4.07)	**0.002**
4^+^ doses	30 (51.7)	28 (48.3)	2.45 (1.29–4.64)	**0.006**
Unknown	543 (76.1)	171 (23.9)	1.12 (0.76–1.64)	0.58
**History of travel to and live in epidemic area**				
Yes	143 (82.7)	30 (17.3)	Ref	
No	733 (71.7)	289 (28.3)	1.56 (1.01–2.41)	0.046

Estimates represent the log odds of “Those who have full or long-term antibodies (antibodies level >  = 0.1 IU/ml)” vs. “Those who have Partial or No antibodies (antibodies level < 0.1 IU/ml)”; aOR: Adjusted OR with multiple variables

**Table 3:** Multiple logistic regression stratified by age group

Variables	Age group					
	**5–15 years**	***p*-value**	**16–25 years**	***p*-value**	**26–40 years**	***p-*value**
	**aOR (95%CI)**		**aOR (95%CI)**		**aOR (95%CI)**	
**Gender**						
Male	Ref		Ref			
Female	0.9 (0.58–1.39)	0.634	1.19 (0.57–2.5)	0.638	1.08 (0.64–1.82)	0.77
**Race**						
Kinh (majority)	2.32 (0.45–12.02)	0.317	3.67 (0.29–47.01)	0.318	1.03 (0.34–3.09)	0.963
Others (ethnic minority)	Ref		Ref			
**Residence**						
Urban area	Ref		Ref			
Rural area	0.86 (0.42–1.77)	0.684	1.18 (0.33–4.3)	0.799	1.31 (0.71–2.42)	0.389
**Income**						
Low income	1.1 (0.5–2.42)	0.806	1.01 (0.27–3.78)	0.985	1.04 (0.5–2.17)	0.918
High/middle income	Ref		Ref			
**Number of people living in one house**						
≤ 4 people	Ref		Ref			
˃ 4 people	1.05 (0.64–1.71)	0.844	1.3 (0.63–2.67)	0.473	0.97 (0.62–1.53)	0.907
**Number of children < 15 living in one house**						
≤ 2 children	Ref		Ref			
˃ 2 children	1.38 (0.71–2.69)	0.338	2.06 (0.38–11.11)	0.401	1.31 (0.68–2.52)	0.418
**Distance from home to the nearest healthcare center**						
< 1 km	Ref		Ref		Ref	
1- < 5 km	1.8 (1.07–3.04)	0.028	0.68 (0.3–1.55)	0.355	1.35 (0.8–2.28)	0.264
≥ 5 km	5.44 (1.16–25.59)	0.032	15.87 (1.53–164.99)	0.021	1.63 (0.55–4.86)	0.382
**Heard about diphtheria**						
Yes	Ref		Ref			
No	2.45 (1.01–5.94)	0.048	0.72 (0.34–1.51)	0.383	1.1 (0.72–1.69)	0.649
**History of diphtheria vaccination**						
1 dose	Ref		Ref		Ref	
2 doses	1.14 (0.56–2.35)	0.714	0.95 (0.17–5.35)	0.957	0.71 (0.21–2.41)	0.58
3 doses	2.33 (1.21–4.49)	**0.011**	3.33 (0.24–46.87)	0.372	1.1 (0.11–11.47)	0.935
4^+^ doses	2.37 (1.14–4.92)	**0.021**	N/A	N/A	3.56 (0.2–63.02)	0.387
Unknown	1.04 (0.54–2)	0.902	1.64 (0.63–4.25)	0.311	0.98 (0.53–1.78)	0.933
**History of travel to and live in epidemic area**						
Yes	Ref		Ref			
No	1.49 (0.59–3.74)	0.4	1.03 (0.41–2.57)	0.951	1.76 (0.96–3.23)	0.067

Estimates represent the log odds of “Those who have full or long-term antibodies (antibodies level >  = 0.1 IU/ml)” vs. “Those who have Partial or No antibodies (antibodies level < 0.1 IU/ml)”; aOR: Adjusted OR with multiple variables

## Discussion

The Expanded Program for Immunization began in Vietnam in 1981. Since 1984, a vaccine containing diphtheria toxoid has been given three doses to children aged 2–4 months, and there are additional doses for 18-month-old children and 7-year-old children in some risk areas [7]. Although the vaccine coverage reported was high [7], Vietnam still has recorded diphtheria cases, and there have been signs of a resurgence in the number of diphtheria cases in recent years [2]. The decline in immunity against diphtheria following vaccination could be a potential reason for the increased incidence of diphtheria in Vietnam [8–10].

In this study, the mean of anti-diphtheria toxoid antibody levels among 1,195 people in the age group of 5 – 40 years was 0.07 (95%CI:0.07–0.08) IU/ml, with 73.3% having partial protection or no protection (< 0.1 IU/ml); only 26.7% had full protection (> = 0.1 IU/ml). This result was in line with the previous study conducted in Nha Trang, Vietnam [8] but this rate was lower compared to 57.8% people having GMC >  = 0.1 IU/ml in Kontum, Vietnam [11], 90.9% in Thailand [12], 76.6% in China [13], 51.4% in Tajikistan [14], but higher compared to 23.5% in Myanmar [15] and 25.8% in Lao [16]. These differences may be explained by differences in vaccination schedules or the characteristics of the study population.

The results of this study indicated a decrease in antibody levels with age. The highest level of antibody was seen in the age group of 5–15 years with 0.09 IU/ml compared to 0.06 IU/ml and 0.07 IU/ml in the age group 16 – 25 years and the age group of 26 – 40 years, respectively. A decreasing trend has also been observed in several previous studies in Vietnam, with a decrease in the antibody level by age despite the vaccination history [8, 17]. A decline in the antibody levels with age has been observed in China [13], Nigeria [18], Myanmar [15], and Australia [19], according to several studies. Following the decline in antibody levels in adolescence and adulthood, some countries are experiencing a re-emergence of diphtheria disease, such as some countries in Europe [20], Nigeria [18], Indonesia [21], Malaysia [22], and Thailand [12].

Antibody levels are associated with history of diphtheria vaccination. A high concentration of antibodies was observed in participants with a history of receiving three or four vaccine doses. Specifically, those who were administered three doses had GMC higher than those two doses or one dose with 0.12 IU/ml compared to 0.07 IU/ml respectively, and who had four doses or more had GMC higher than those received three doses with 0.13 IU/ml compared to 0.12 IU/ml, respectively. There is an association between antibody level category and the vaccination history; those who had three or 4^+^ doses had antibody level two times higher than those who had one dose, and this association has a significant difference. This was more obvious in the age group 5 – 15 years with those who had three or 4^+^ doses being approximately 2.3 times higher antibody level than those who had one dose, and this association was significantly different. A similar association was observed in an interventional study conducted in Kon Tum, Vietnam, where there was an increase in antibody levels following vaccination [10] and vaccinated people in the past 10 years had higher immunity than those who had no or unknown vaccination [11]. A study in Australia also revealed that elderly individuals who received single-shot vaccinations for tetanus and diphtheria at 5-year intervals did not develop long-term immunity against diphtheria [23]. In addition, diphtheria cases have been observed in individuals with a history of partial immunization [24] or unvaccinated [25], and the majority of cases were observed in adolescents and adults [18, 21]. According to the guidelines of the Ministry of Health in Vietnam regarding the EPI to prevent and control diphtheria, children under one year of age require three primary doses of a vaccine containing a diphtheria component. A booster dose should be administered at 18–24 months of age (dose 4), another at 4–7 years of age (dose 5), a final dose at 9–15 years of age (dose 6), and a booster dose every 10 years thereafter [26]. However, only 12.3% of participants were administered a vaccine containing a diphtheria component at three or more doses based on a confirmed vaccination record. In addition, the majority of people who were administered three or more booster doses were from the age group 5–15 years, whereas less proportion of of them belonged to the age group 16 – 25 years and 26 -40 years. Several studies have reported low vaccine coverage with booster doses [27, 28]. This raises concerns regarding vaccine coverage, especially regarding the booster doses for adolescents and adults. The low antibody levels in adolescents and adults in Khanh Hoa province could be explained by the low booster dose coverage in adolescents and adults, which raises the risk of a diphtheria outbreak in a community with more than 1.3 million people due to an increase in susceptible people. In addition, the majority of unknown vaccination statuses fall in the age group of 16 years or above since the personal vaccine record is no longer maintained. Other studies with similar findings recommend booster doses of the diphtheria containing vaccine to adolescents and adults to maintain the level of immunity in the population to school-age children in the age group 6 to 17 years [29], to adults in the age group 20–50 years [27] or the age group of 30 – 64 years [30] or booster doses for the adults every 10 years [11, 31]. Policymakers in Khanh Hoa province should consider implementing more school-based vaccination programs for children to receive their booster doses during the school year. Additionally, when it comes to providing booster doses to adults, policymakers should introduce targeted adult immunization campaigns, possibly in collaboration with workplaces in high-risk areas, to reach this age group effectively.

The multiple logistic regression model demonstrated that some factors were significantly associated with the antibody level, such as age, history of vaccination, and distance from home to the nearest healthcare center. Previous studies in Vietnam demonstrated no significant differences in seroprotection prevalence according to sex, ethnicity, or residence [8, 11]. However, the study in Turkey shows that the GMC in males was significantly higher than in females [28] and the study in Lao has results that females older than 16 years of age have GMC significantly higher than males ≤ 13 years of age [16]. There seems to be no clear difference in antibody levels between the sexes, and the antibody level of each population depends on the vaccine schedule and the response of the immune system to the vaccine. The explanation of this term requires further investigation. Moreover, the factors like hearing about diphtheria before showed no significant difference, but an important point is that, the proportion of people who had ever heard about diphtheria was very low (only 6.9%). This indicates the importance of risk communication for diphtheria prevention.

This study has some limitations as the bias in memory recall of participant regarding their immunization history. Some selection bias may have been introduced in the multiple stage selection methods based on probability proportional to size.

## Conclusion

Regarding the decrease of the anti-diphtheria toxoid antibody level with age and as we found in this study, it is important to maintain a protective level of antibodies to prevent outbreaks of diphtheria in Vietnam. First, it is crucial to promote routine vaccination coverage to over 95% in the EPI for children and further booster doses of diphtheria toxoid containing vaccine through adulthood. According to guidelines from the WHO, booster doses of vaccines should be administered at specific ages: 12–23 months (dose 4), another at 4–7 years (dose 5), and a final dose at 9–15 years (dose 6). It is also recommended that adults receive booster doses at least every 10 years. This schedule ensures a reduced risk of diphtheria epidemics.

### Supplementary Information

Supplementary Material 1.

## Data Availability

The datasets used and/or analysed during the current study are available from the corresponding author on reasonable request.

## Abbreviations

CI: Confidence Interval
DPT: Diphtheria, Pertussis, Tetanus
ELISA: Enzyme-Linked Immunosorbent Assay
GMC: Geometric mean concentrations
IgG: Immunoglobulin G
OR: Odds Ratio
PPS: Probability Proportional To Size
Td: Tetanus, diphtheria
WHO: World Health Organization
