# Supplementary material for: Seroprevalence of anti-diphtheria toxoid antibody and implications for vaccination policy in Vietnam’s South-central coast: a cross-sectional study
Source: BMC Infect Dis. 2024 Aug 12;24:813. doi: 10.1186/s12879-024-09688-0 (PMC11318120; doi:10.1186/s12879-024-09688-0)
Supplement: Supplementary file 1 — Supplementary Material 1. [file 12879_2024_9688_MOESM1_ESM.pdf]

Supplementary

Selection of communes/wards

Step 1: List of all communes/wards in 8 districts in Khanh Hoa province with population aged 5 - 40 years in each commune/ward

Step 2: Calculate the culmulative population for each commune/ward

Step 3: Divide the total population by the number of clusters to be sampled (30 communes/wards), to get the Sampling Interval (SI)

Step 4: Choose a random number between 1 and the SI. The first cluster to be sampled contains this cumulative population

Step 5: Calculate the following series: RS; RS + SI; RS + 2SI; …. RS+(d-1)*S reaching a total of 30 clusters

Selection of participants

Step 6: In each clusters mentioned above, select 01 subgroup using simple random sampling method

Step 7: In each subgroup, randomly select 01 residential cluster. Within the central area of the residential cluster, choose one direction for conducting the survey using simple random sampling method

Step 8: Along the chosen survey direction, assign sequential numbers to the houses on that direction from 1 to 10. The "first house" to conduct the survey will be randomly selected from the numbers 1-10. The "second house" and subsequent houses will be selected using the "next-door neighbor" method, turning right

Step 9: Each household selects only 2 individuals who meet the criteria of the research subjects by using randomly selected from the list of household members aged 5 - 40 based on increasing order of age

Figure S1. Flow chart of Probability Proportional to Size (PPS)

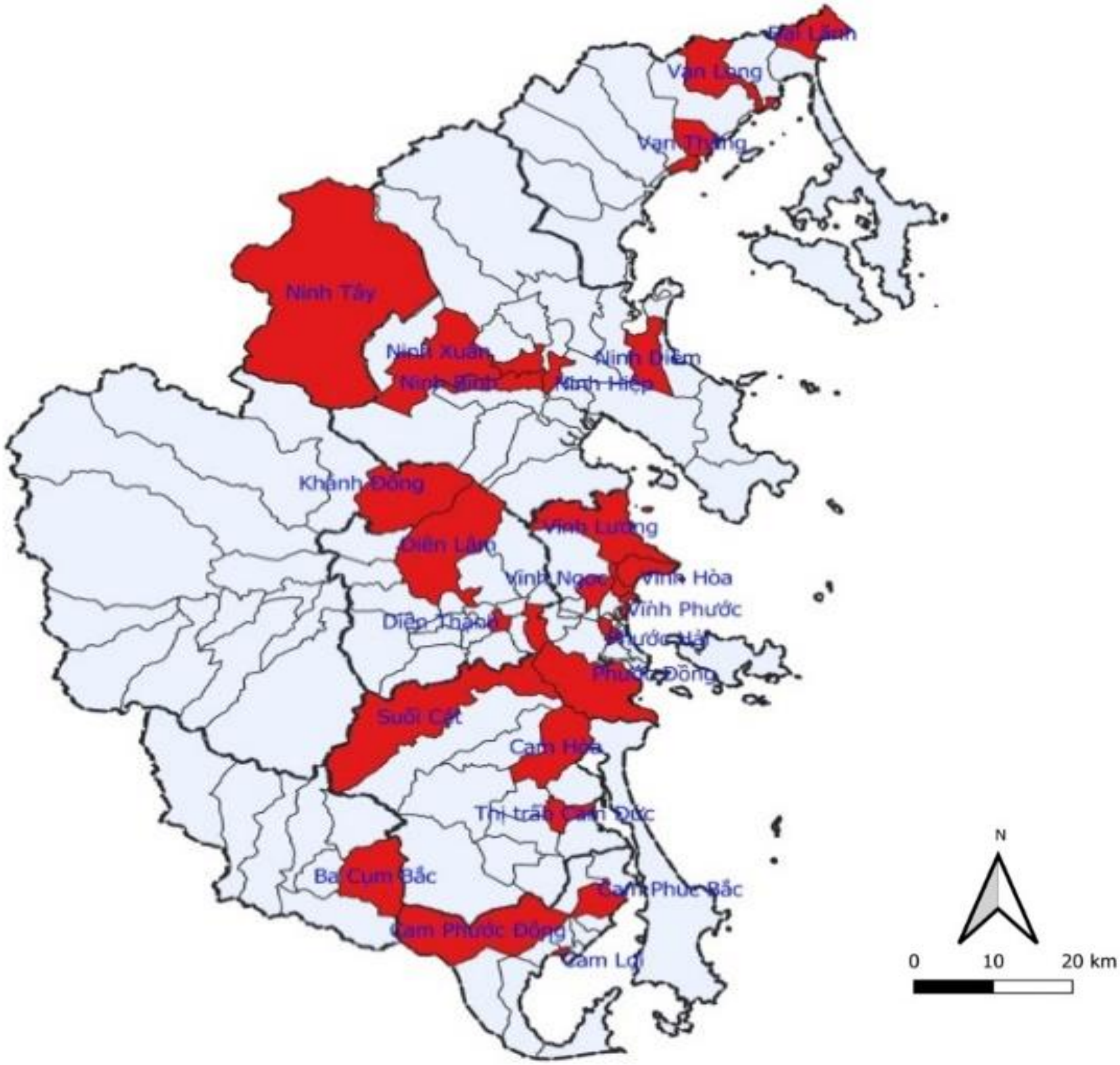

Figure S2. Study area in Khanh Hoa province
